# Thymoma with Extensive Necrosis: A Case Report and Literature Review

**DOI:** 10.70352/scrj.cr.25-0152

**Published:** 2025-06-18

**Authors:** Yuki Katsumata, Shigeki Suzuki, Yuki Ishiguro, Hiroyuki Sakamaki, Akio Kazama, Keisuke Asakura

**Affiliations:** 1 Department of General Thoracic Surgery, Sagamihara Kyodo Hospital, Sagamihara, Kanagawa, Japan; 2 Division of Thoracic Surgery, Department of Surgery, Keio University School of Medicine, Tokyo, Japan; 3 Division of Pathology, Sagamihara Kyodo Hospital, Sagamihara, Kanagawa, Japan

**Keywords:** mediastinal tumor, thymoma, necrosis

## Abstract

**INTRODUCTION:**

Thymomas are associated with necrosis and hemorrhage in approximately 5% of cases; however, cases in which necrotic tissue constitutes the majority of tumors are rare.

**CASE PRESENTATION:**

A 30-year-old man was referred to our hospital with transient anterior chest pain and a mediastinal mass detected on a health check-up. Positron emission tomography/computed tomography showed no fluorodeoxyglucose uptake, and serum and biochemical analyses revealed no elevated inflammatory responses or tumor marker levels. Based on imaging findings, thymic cysts and thymomas were considered differential diagnoses, and thoracoscopic mediastinal tumor resection was performed. The encapsulated tumor, which was adherent to the lung via the thickened pleura, was successfully resected, and a rapid diagnosis of thymoma was made. Final pathological examination confirmed a type B2 thymoma, with necrosis comprising approximately 80% of the tumor. The patient has been followed up on an outpatient basis, with no recurrence at 1 year after surgery.

**CONCLUSIONS:**

Extensive necrosis in thymic tumors often suggests high-grade malignancy, but may also occur in necrotic thymoma with a favorable prognosis. Recognizing this possibility is essential to avoid overtreatment and guide appropriate surgical management.

## Abbreviations

AchR
: acetylcholine receptor
AFP
: alpha-fetoprotein
CD
: cluster of differentiation
CEA
: carcinoembryonic antigen
CRP
: C-reactive protein
HCG
: human chorionic gonadotropin
sIL-2R
: soluble interleukin-2 receptor
SUV
: standardized uptake value
WBC
: white blood cell
WHO
: World Health Organization

## INTRODUCTION

Although 5% of thymomas are associated with necrosis, hemorrhage, or cyst formation in the tumor tissue,^1,2)^ cases in which necrotic tissue occupies the majority of the tumor are rare and are described as necrotic thymoma.^3)^ To date, only 12 cases of necrotic thymoma have been reported in Japan (**Table 1**).^2–13)^ This report describes a case of resected necrotic thymoma at our hospital.

**Table 1:** Previous literature on thymoma with extensive necrosis

Case	Author	Year	Age	Sex	Syncope		Size (mm)	SUVmax	Masaoka staging	WHO classification	Observation period (m)
					Fever	Chest pain					
1	Present case	2024	29	M	+	+	50	None	I	Type B2	12
2	Hinokuma^3)^	2020	36	M	+	+	60	N.A.	I	Type B2	12
3	Ike^4)^	2017	41	M	+	+	39	N.A.	I	Type B1	12
4	Jang^5)^	2015	70	F	−	+	40	N.A.	I	Type B1	10
5	Kataoka^2)^	2015	52	F	+	+	55	3.1	I	Type B1	24
6	Himuro^6)^	2014	73	F	−	+	65	N.A.	I	Type B2	72
7	Yamashita^7)^	2013	40	M	+	−	85	N.A.	II	Type B2/3	N.A.
8	Takasaki^8)^	2012	46	M	+	+	75	6.0	II	Type B2	12
9	Hori^9)^	2008	38	M	+	+	35	N.A.	II	Type B2	9
10	Itoh^10)^	2006	25	M	+	+	90	N.A.	I	Type B1	24
11	Ishibashi^11)^	2003	44	F	+	+	50	N.A.	I	Type B2	36
12	Kataoka^12)^	1994	50	F	−	+	58	N.A.	I	Type B1/2	N.A.
13	Hayashi^13)^	1993	44	M	+	+	40	N.A.	I	Type B2	N.A.

F, female; M, male; N.A., not applicable; SUV, standardized uptake value; WHO, World Health Organization

## CASE PRESENTATION

A 30-year-old man was referred to our hospital with a mediastinal mass detected on chest radiography during a health check-up. The patient had no significant medical history but reported smoking 20 cigarettes per day for 10 years. He had experienced sharp anterior chest pain 1 month prior; however, the pain had resolved by the time of his visit to our hospital. On physical examination, the patient’s body temperature was 36.7°C, percutaneous oxygen saturation was 100% in room air, and there was no evidence of myasthenia gravis. Laboratory findings on admission were as follows: CRP, 0.15 mg/dL; WBC, 5700/μL; CEA, 1.5 ng/mL; AFP, 1.3 ng/mL; HCG, <0.2 mIU/mL; sIL-2R, 150 U/mL; and anti-AchR antibody <0.2 nmol/L.

**Fig. 1:**
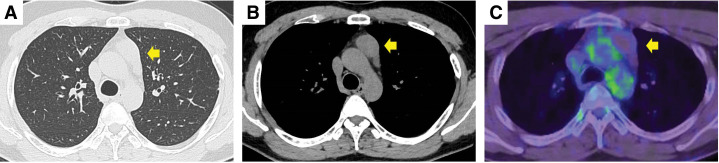
Preoperative image. Chest computed tomography shows a 35 × 25 mm mass on the left side of the anterior mediastinum in the lung (**A**: arrow) and mediastinal windows (**B**: arrow). Positron emission tomography with 18-F-fluorodeoxyglucose demonstrated no accumulation in the mass (**C**: arrow).

A preoperative chest radiography revealed a mass shadow overlapping the ascending aorta. Plain chest CT showed a 35 × 25 mm mass on the left side of the anterior mediastinum (**Fig. 1A** and **1B**). Contrast-enhanced CT was not performed due to the patient’s iodine allergy, and MRI was omitted because of financial constraints.

**Fig. 2:**
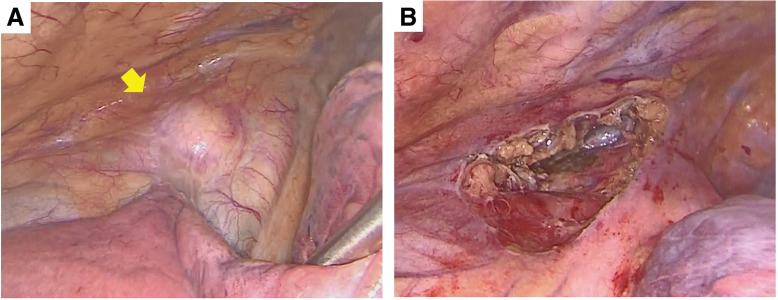
Intraoperative image. Thoracoscopy revealed a 30-mm-sized mass in the anterior mediastinum (arrow) with mild fibrous adhesions to the lung (**A**). The mass was resected along with as much surrounding thymic tissue as possible (**B**).

**Fig. 3:**
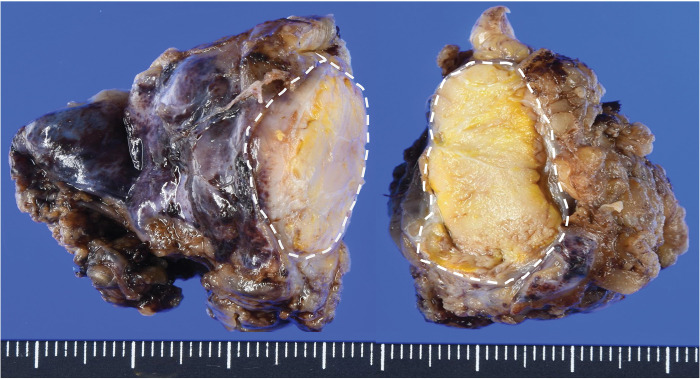
Pathological findings (the cut surface). The cut surface of the surgical specimen measured 50 mm in diameter and 30 mm in width, exhibiting a uniform yellowish-white coloration. The tumor area is demarcated by a white dashed line. The areas outside the dashed lines represent thymic tissue.

**Fig. 4:**
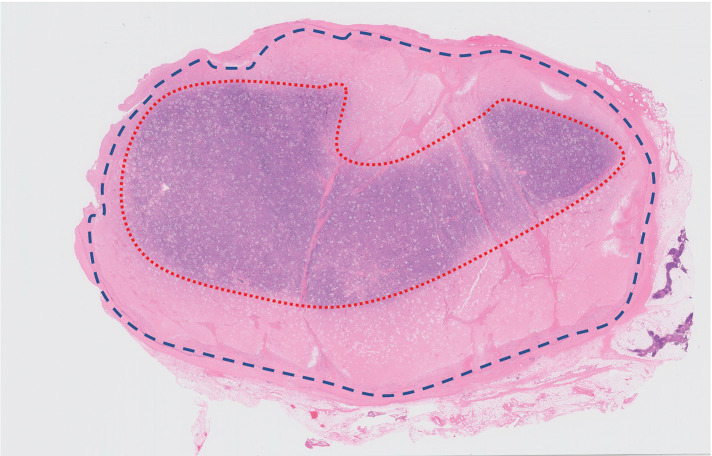
Pathological findings (macroscopic image). Macroscopic finding (loupe image) of the largest segment of the resected tumor. The tumor capsule is outlined by a dashed line, and a dotted line marks the basophilic region, which is visually distinguishable. Viable thymic epithelial cells were identified within the basophilic region.

**Fig. 5:**
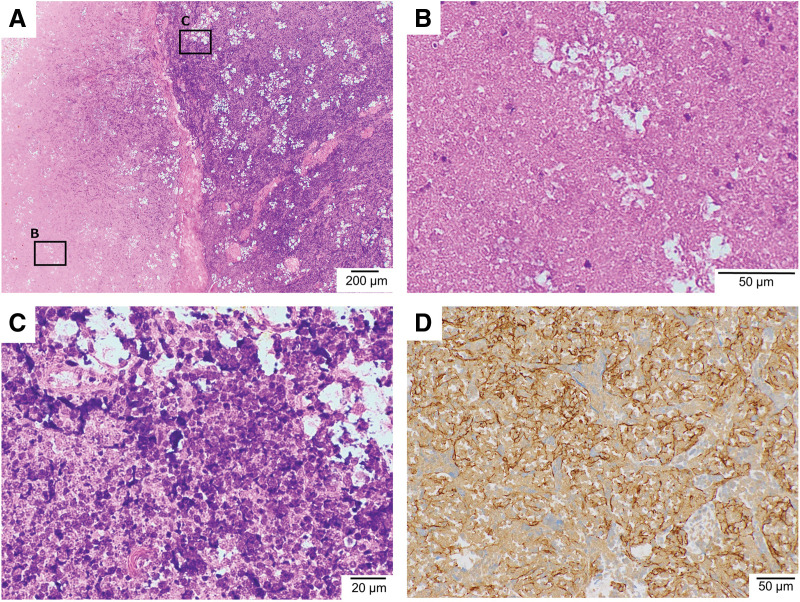
Pathological findings (microscopic image). The magnified image illustrates the boundary between the acidophilic and basophilic regions in **Fig. 3** (**A**). In the HE-stained acidophilic region, thymic epithelial cells exhibited signs of coagulation necrosis, characterized by cytoplasmic acidification and nuclear enrichment and loss (**B**). HE staining also revealed basophilic regions containing poorly atypical thymic epithelial cells, along with aggregates of lymphocytes and a reticulated arrangement. Within the basophilic region, necrotic changes were noted in a worm-eaten pattern, with residual thymic tissue appearing as islands (**C**). Immunohistochemical analysis demonstrated the presence of CD3-positive T cells and reticulated thymic epithelial cells in the residual thymic tissue (**D**). HE, hematoxylin and eosin

PET/CT revealed no fluorodeoxyglucose (FDG) accumulation in the mass, and no obvious lymph node or distant metastases were detected (**Fig. 1C**). Based on blood samples and imaging findings, thymic cysts and thymomas were considered the primary differential diagnoses, and video-assisted mediastinal tumor resection was performed (**Fig. 2A** and **2B**). The patient was approached from the left thoracic cavity in the right lateral recumbent position with a total of 3 ports: a 10 mm port at the mid-axillary line of the 6th intercostal space, a 30 mm port at the anterior axillary line of the 4th intercostal space, and a 5 mm port at the posterior axillary line. Thoracoscopy revealed a 30-mm-sized mass in the anterior mediastinum with mild membranous fibrous adhesions to the lungs (**Fig. 2A**). The adhesions were easily detached using a cotton swab, and no tumor invasion was observed. The tumor was an elastic, firm mass with cystic components, suggestive of a thymoma with cystic changes. The mass, which had a thickened inflammatory capsule, was resected along with as much surrounding thymic tissue as possible (**Fig. 2B**). Rapid intraoperative diagnosis identified the tumor as a thymoma with extensive necrosis. The operation time was 1 hour and 10 minutes, with minimal bleeding. Gross examination of the excised specimen revealed a 50 × 30 mm oval tumor with a uniform yellowish-white color on the fractured surface (**Fig. 3**). The tumor area is outlined by a dashed line in **Fig. 3**, with the surrounding thymic tissue outside this boundary. Histopathological findings (loupe image: **Fig. 4**) showed the tumor capsule demarcated by a dashed line, and a basophilic area distinguishable by the naked eye, marked by a dotted line. In the microscopic image (**Fig. 5**), the boundary between the acidophilic and basic areas in **Fig. 3** was partially enlarged (**Fig. 5A**). In the highly acidophilic regions of hematoxylin and eosin staining, thymic epithelial cells exhibited evidence of coagulation necrosis with cytoplasmic eosinophilia and nuclear enrichment and loss (**Fig. 5B**). Necrotic tissue was also observed within this acidophilic region, whereas viable thymic tissues were observed as islands (**Fig. 5C**). Immunohistochemistry demonstrated CD3-positive T cells and a reticular arrangement of thymic epithelial cells (**Fig. 5D**). The acidophilic area at the tumor margin in **Fig. 4** consisted primarily of necrosis, whereas the basophilic area in the center also showed extensive necrosis. This case was diagnosed as WHO type B thymoma, with minimal cytological atypia in the thymic epithelial cells. The patient remained asymptomatic and showed no evidence of recurrence during 1.5 years of follow-up.

## DISCUSSION

Thymoma with extensive necrosis represents a rare pathological variant whose clinical and histological characteristics remain incompletely understood. Although tumor necrosis is generally considered a poor prognostic factor in many malignancies,^14,15)^ this may not apply uniformly to thymic epithelial tumors.^1)^ Specifically, thymic carcinoma is generally recognized as a high-grade malignancy with poor prognosis and is frequently characterized by extensive hemorrhage and necrosis.^16)^ However, some thymomas, despite exhibiting extensive coagulative necrosis, may follow a benign clinical course, as demonstrated in previous reports.^2–13)^

The distribution of necrosis in our case is notably atypical. As illustrated in **Fig. 4**, necrosis was predominantly observed at the tumor periphery, whereas viable thymic epithelial tissue was relatively preserved in the central region. This pattern contrasts with the central necrosis commonly reported in previous cases of necrotic thymoma, which has generally been attributed to ischemia resulting from rapid tumor growth that outpaces its central blood supply.^1–3)^ Other mechanisms that have been proposed for tumor necrosis include thrombotic occlusion of intratumoral vessels and immune-mediated injury. In particular, peripheral necrosis has been hypothesized to arise from immune responses occurring at the tumor margins, where lymphocytic infiltration is often most pronounced, especially in type B thymomas.^17)^ However, in our case, histopathological analysis revealed neither thrombotic vessels nor conspicuous immune cell infiltration at the necrotic sites. In the absence of definitive evidence for either vascular occlusion or active immune-mediated cytotoxicity, the etiology of the peripheral-dominant necrosis remains unclear. Further pathological and immunological investigations in additional cases are warranted to clarify the underlying mechanisms of this unusual pattern of necrosis.

Regarding the clinical implications of necrosis in thymomas, it is essential to distinguish between thymomas with necrosis, those without necrosis, and thymic carcinomas. Thymic carcinomas are histologically characterized by marked cytological atypia, increased mitotic activity, and destruction of the lobular architecture.^16)^ In contrast, thymomas—whether necrotic or not—tend to retain some elements of lobulated structure and exhibit less pronounced atypia.^1)^ Immunohistochemical profiling aids in this differentiation: thymic carcinomas often express CD5 and CD117,^16)^ whereas thymomas generally do not. In our case, the retention of lobular architecture and the presence of CD3-positive T-cell infiltrates within the residual thymic tissue supported the diagnosis of necrotic thymoma rather than thymic carcinoma. Some reports suggest that PET/CT imaging is useful for differentiating thymic carcinoma from thymoma.^18,19)^ Although it is difficult to distinguish high-grade invasive thymoma from thymic carcinoma based on SUV values alone, the absence or near absence of FDG uptake strongly suggests a low-grade tumor. Notably, the presence of extensive necrosis in thymoma does not appear to be associated with a worse prognosis.^1,2)^ Among the 13 reported cases of necrotic thymoma in Japan, none showed recurrence during the follow-up period.^2–13)^ This contrasts with the well-established correlation between tumor necrosis and poor prognosis in other solid malignancies, such as pleomorphic carcinoma of the lung^20)^ or hepatocellular carcinoma.^21)^ Furthermore, the existence of spontaneously regressing thymomas, some of which display histological necrosis, raises the possibility that necrosis may reflect a form of immune-mediated tumor regression.^22–24)^ Thus, thymoma-associated necrosis might represent a fundamentally different biological process than that observed in other cancers.

## CONCLUSIONS

Extensive necrosis in thymic tumors often suggests high-grade malignancy, but it may also occur in necrotic thymoma with a favorable prognosis. Recognizing this possibility is essential to avoid overtreatment and guide appropriate surgical management.

## ACKNOWLEDGMENTS

We would like to thank Editage (www.editage.jp) for the English language editing.

## DECLARATIONS

### Funding

No external funding was received for this study, including funding for equipment or medications.

### Authors’ contributions

YK and SS contributed to the acquisition of clinical data and prepared the manuscript for this case report.

SS performed the surgery and perioperative care and contributed to the drafting and revising of the manuscript.

YI, HS, AK, and KA supervised clinical decision-making, implemented the treatment plan, and contributed to manuscript preparation.

All authors have read and approved the final version of this manuscript.

### Availability of data and materials

Not applicable.

### Ethics approval and consent to participate

Not applicable.

### Consent for publication

Written informed consent was obtained from the patient for publication of this report.

### Competing interests

The authors declare that they have no competing interests.
